# Food Insecurity and Cardiometabolic Risk Factors in Adolescents

**DOI:** 10.5888/pcd14.170222

**Published:** 2017-11-09

**Authors:** Shannon M. Robson, Alicia J. Lozano, Mia Papas, Freda Patterson

**Affiliations:** 1 Department of Behavioral Health and Nutrition, College of Health Sciences, University of Delaware, Newark, Delaware; 2 School of Nursing, University of Pennsylvania, Pennsylvania; 3 Value Institute, Christiana Care Health System, Newark, Delaware

## Abstract

**Introduction:**

Food insecurity is associated with poor cardiometabolic health in adults. The extent to which this relationship exists in adolescents has yet to be defined. The objective of this study was to examine the relationship between food insecurity and cardiometabolic risk factors in adolescents.

**Methods:**

We evaluated the association between food insecurity and several cardiometabolic risk factors by using data collected from the Youth Risk Behavior Survey at the state and city levels. Logistic regression models adjusted for sex, race/ethnicity, grade, and neighborhood safety were used to determine the association between food insecurity and cardiometabolic risk factors among a weighted sample of 495,509 adolescents.

**Results:**

Of the sample studied, 12.8% reported being food insecure. Food-insecure adolescents had more than a twofold increased odds of not eating breakfast on all 7 days (adjusted odds ratio [AOR] = 2.27; 95% confidence interval [CI], 1.61–3.21; *P* < .001), a 60% increased odds of reporting less than 8 hours per day of sleep (AOR = 1.60; 95% CI, 1.15–2.23; *P* = .006), a 65% increased odds of reporting current cigarette smoking (AOR = 1.65; 95% CI, 1.16–2.36; *P* = .006), and a 65 % increased odds of current alcohol consumption (AOR = 1.36; CI, 1.01–1.84; *P* = .04), compared with food-secure adolescents.

**Conclusion:**

Among adolescents, in adjusted models, food insecurity was significantly associated with not consuming breakfast daily, getting less than 8 hours of sleep per day, currently smoking, and currently drinking alcohol. Food insecurity in adolescents may serve as an important precursor to poor cardiometabolic health.

## Introduction

Food insecurity remains a critical public health concern. As many as 1 in 6, approximately 16.6%, of households with children in the United States do not have access to enough food for an active, healthy life (1). Poor cardiovascular and metabolic outcomes are among the range of negative health outcomes associated with food insecurity in adults (1–3). If strong relationships exist, screening for food insecurity early in life may provide helpful information to help curb the growing prevalence of cardiometabolic disease.

Food-insecure adolescents are an overlooked segment of the food-insecure population; however, most research and programming efforts have been directed at adults and young children. Nevertheless, adolescence is a life stage defined by physical, social, and psychological development where autonomous choices about engagement in health behaviors are formed and often continue into adulthood (4). Quantifying the extent to which food insecurity in adolescents is related to a broad range of cardiometabolic risk behaviors (eg, physical inactivity [5], sedentary behavior [6], sleep duration [7], breakfast-skipping [8]) could help inform primary prevention approaches to offsetting cardiometabolic disease outcomes in adulthood. To date, few investigations have been made into food insecurity and cardiometabolic risk behaviors in adolescents and most have been confined to dietary behaviors and obesity outcomes (9–12). We examined the association of food security status (food secure vs food insecure) with a range of cardiometabolic risk factors in a representative statewide sample of Pennsylvania adolescents. Considering these established relationships in adults, the objective of this study was to examine the relationship between food insecurity and cardiometabolic risk factors in adolescents.

## Methods

We used data from the Youth Risk Behavior Survey (YRBS) collected in the state of Pennsylvania during the 2014–2015 school year. The YRBS is a self-administered, multiple-choice survey of US high school students that monitors 6 categories of priority health-risk behaviors: behaviors relating to injuries and violence, sexual risk behaviors, tobacco use, alcohol and other drug use, unhealthy diet, and physical inactivity (13). The survey is administered biennially by the Centers for Disease Control and Prevention and includes representative samples of students in grades 9 through 12 by using a 2-stage–cluster sample design. A student response rate of at least 60% is required to achieve weighted data that are representative of the population. For our analysis, we surveyed 64 high schools from nonurban regions of Pennsylvania, and received an 80% (2,899 of 3,631) student response rate. Twenty-nine high schools from the urban center of Philadelphia were surveyed, and had a 65% (1,896 of 2,909) student response rate. The total study population of 6,540 students consisted of 150 primary sampling units (PSUs) in 32 strata (an average of 40 observations per PSU), and is weighted to a population estimate of 542,799 accounting for the complex sample design. Adolescents with complete data (N = 4,994) were used in this analysis, giving us a final weighted sample of 495,509. Parent permission was received according to local procedures before administration of the survey. This study was approved by the Temple University institutional review board (protocol no. 21251).

The outcomes of interest were several cardiometabolic risk factors (dietary intake, overweight/obesity, screen time, physical activity, sleep, and substance use). The following variables of dietary intake, overweight/obesity, activity, and sleep were evaluated: daily consumption of breakfast (on ≥7 d vs <7 d) (14), fruit and vegetable consumption (≥5 servings/d vs <5 servings/d), soda consumption (≥1 time/d vs <1 time/d), and milk consumption (≥1 time/d vs <1 time/d). Participant overweight or obesity status was evaluated by using body mass index (BMI, kg/m^2^) on the basis of self-reported weight height; age-specific and sex-specific BMI percentiles were computed (15). Students whose BMI was at or above the 95th percentile were classified as obese; students whose BMI was in the 85th through 94th percentile were classified as overweight (15).

Physical activity levels were evaluated as a single item that asked students on how many days of the past 7 days they were physically active for at least 60 minutes; responses were dichotomized to 0 through 6 days versus 7 days, on the basis of national recommendations for adolescents to obtain at least 60 minutes of physical activity per day (16). Sedentary behavior was measured as average hours per day of television viewing and recreational computer use, combined; responses were dichotomized on the basis of the median split of less than 3 hours per day versus 3 hours or more per day. Sleep duration was measured as the number of hours of sleep the respondent got on the average school night (≤4 h, 5 h, 6 h, 7 h, 7, 8 h, 9 h, ≥10 h); responses were dichotomized as 8 or more hours per night versus less than 8 hours per night.

Substance use variables of current cigarette smoking and alcohol use were also examined. Respondents who reported smoking one or more cigarettes in the past month were coded as current smokers, and respondents who consumed one or more alcoholic beverages in the past month were considered current alcohol consumers.

The predictor of interest was food insecurity. This was assessed by using a single item that asked respondents, “During the past 30 days, how often did you go hungry because there was not enough food in your home?” Responses were on an ordinal scale to include never, rarely, sometimes, most of the time, and always. For this analysis, food-insecure adolescents were those who responded always, most of the time, or sometimes, and food-secure adolescents were those who responded rarely or never (17).

Covariates included the sociodemographic variables of age in years (12–13 y, 14 y, 15 y, 16 y, 17 y, ≥18 y), sex (male vs female), race/ethnicity (Asian, non-Hispanic black, non-Hispanic white, Hispanic, mixed/other), grade (9th, 10th, 11th, 12th, other/ungraded), and neighborhood safety, where respondents indicated the extent to which they felt safe and secure in their neighborhood (always or most of the time vs never, rarely, or sometimes).

We used descriptive statistics (percentages and 95% confidence intervals [CIs]) to characterize all variables, overall, and by food security status (food secure and food insecure). To quantify the association between food insecurity with several cardiometabolic risk factors, we generated univariate logistic regression models. All models were then adjusted for sex, race/ethnicity, school grade, and neighborhood safety. Unadjusted and adjusted odds ratios (ORs) and their 95% CIs were calculated. The study’s complex survey sampling method was taken into account, and sampling weights were used in all analyses. Significance was set at an α level of .05. Because of the number of outcome variables, only significant ORs (≥1.5) are discussed. All analyses were conducted using SPSS Statistics, version 24 (IBM Corp).

## Results

Overall, 87.2% of the 495,509 adolescents in our sample reported being food secure, and the remaining 12.8% were food insecure. Most adolescents were non-Hispanic white (69.9%) and felt safe and secure in their neighborhood (83.7%). Most had poor dietary habits: 65.1% did not consume breakfast daily, 87.3% ate fewer than 5 servings of fruits and vegetables per day, 18.1% drank sodas 1 or more times per day, and 62.2% reported drinking milk less than once a day. In terms of activity and sleep variables, 29.8% were overweight or obese and most (75.2%) were not physically active or did not sleep 8 or more hours on school nights (74.6%). Most (87.9%) did not currently smoke cigarettes or drink alcohol (69.5%) (Table 1).

**Table 1:** Sociodemographic and Cardiometabolic Risk Factors of Pennsylvania Adolescents, by Food-Security Status, Youth Risk Behavior Survey, 2014–2015^a^

Variable	Total Sample (n = 495,509)	Food Secure (n = 432,117)	Food Insecure (n = 63,392)
**Age, y**			
12–13	0.2 (0–1.0)	0.2 (0–1.2)	0.1 (0–0.3)
14	15.7 (11.4–21.3)	15.6 (11.2–21.4)	16.2 (11.0–23.3)
15	24.4 (20.2–29.1)	25.1 (20.7–30.1)	19.5 (14.8–25.2)
16	26.5 (22.5–31.0)	26.6 (22.4–31.4)	26.0 (21.0–31.7)
17	22.1 (18.8–25.9)	21.8 (18.5–25.4)	24.7 (18.7–31.8)
≥18	11.0 (8.4–14.4)	10.7 (8.1–14.0)	13.5 (9.4–19.2)
**Sex**			
Female	49.8 (47.5–52.2)	49.7 (47.2–52.3)	50.7 (43.7–57.7)
Male	50.2 (47.8–52.5)	50.3 (47.7–52.8)	49.3 (42.3–56.3)
**Grade**			
9	25.4 (19.1–32.9)	25.7 (19.2–33.4)	23.3 (16.9–31.2)
10	25.2 (19.5–31.8)	25.1 (19.2–32.2)	25.3 (19.4–32.2)
11	24.5 (19.6–30.1)	25.0 (19.9–30.9)	21.1 (15.3–28.2)
12	24.6 (19.5–30.6)	23.9 (18.9–29.7)	29.6 (21.1–39.9)
Ungraded/other	0.4 (0.1–1.7)	0.3 (0.1–1.7)	0.7 (0.2–2.4)
**Race/ethnicity**			
Asian	2.5 (1.8–3.3)	2.3 (1.7–3.2)	3.5 (2.0–6.0)
Non-Hispanic black	16.0 (11.3–22.2)	15.2 (10.5–21.5)	21.6 (15.5–29.4)
Non-Hispanic white	69.9 (62.7–76.2)	71.5 (64.3–77.7)	59.3 (50.0–67.9)
Hispanic	8.4 (6.3–11.1)	7.9 (5.8–10.6)	12.2 (8.7–16.7)
Mixed/other	3.2 (2.7–3.8)	3.2 (2.6–3.8)	3.4 (2.2–5.3)
**Neighborhood safety (feel safe and secure)**			
No	16.3 (13.6–19.4)	14.0 (11.5–16.9)	32.3 (25.5–39.8)
Yes	83.7 (80.6–86.4)	86.0 (83.1–88.5)	67.7 (60.2–74.5)
**Daily breakfast consumption**			
No	65.1 (61.9–68.1)	62.8 (59.4–66.0)	80.6 (75.0–85.2)
Yes	34.9 (31.9–38.1)	37.2 (34.0–40.6)	19.4 (14.8–25.0)
**Fruit and vegetable consumption (≥5 servings per day)**			
No	87.3 (85.7–88.8)	87.1 (85.3–88.7)	88.6 (84.5–91.8)
Yes	12.7 (11.2–14.3)	12.9 (11.3–14.7)	11.4 (8.2–15.5)
**Soda consumption (≥1 time per day)**			
No	81.9 (79.5–84.1)	82.5 (80.1–84.6)	78.0 (72.0–83.1)
Yes	18.1 (15.9–20.5)	17.5 (15.4–19.9)	22.0 (16.9–28.0)
**Milk consumption (≥1 time per day)**			
No	62.2 (59.2–65.0)	62.1 (59.3–64.9)	62.6 (56.6–68.2)
Yes	37.8 (35.0–40.8)	37.9 (35.1–40.7)	37.4 (31.8–43.4)
**Overweight/obese**			
No	70.2 (67.9–72.4)	70.7 (68.1–73.1)	67.0 (62.0–71.8)
Yes	29.8 (27.6–32.1)	29.3 (26.9–31.9)	33.0 (28.2–38.0)
**Physical activity, 60 min/day on all 7 days**			
No	75.2 (72.8–77.5)	75.3 (72.9–77.6)	74.8 (69.3–79.7)
Yes	24.8 (22.5–27.2)	24.7 (22.4–27.1)	25.2 (20.3–30.7)
**Screen time, hours**			
<3	42.9 (40.7–45.1)	43.8 (41.3–46.3)	36.8 (30.1–44.0)
≥3	57.1 (54.9–59.3)	56.2 (53.7–58.7)	63.2 (56.0–69.9)
**Sleep (≥8 h/d)**			
No	74.6 (72.3–76.8)	73.4 (70.9–75.8)	83.0 (78.6–86.7)
Yes	25.4 (23.2–27.7)	26.6 (24.2–29.1)	17.0 (13.3–21.4)
**Currently smoke cigarettes**			
No	87.9 (85.4–90.0)	88.8 (86.4–90.8)	81.4 (75.9–85.8)
Yes	12.1 (10.0–14.6)	11.2 (9.2–13.6)	18.6 (14.2–24.1)
**Currently drink alcohol**			
No	69.5 (66.4–72.5)	70.4 (67.5–73.2)	63.1 (56.0–69.7)
Yes	30.5 (27.5–33.6)	29.6 (26.8–32.5)	36.9 (30.3–44.0)

Abbreviations: CI, confidence interval; OR, odds ratio.

a Values are percentage (95% confidence interval).

Univariate logistic regression models demonstrated that food insecurity was significantly associated with not eating breakfast daily, insufficient sleep, current cigarette smoking, and current alcohol consumption. Food-insecure adolescents had nearly a 2.5-fold increased odds of not eating breakfast daily compared with food-secure adolescents (OR = 2.46; 95% CI, 1.75–3.47; *P* < .001) (Table 2). Compared with food-secure adolescents, those who were food insecure had a 77% increased odds of reporting less than 8 hours per day of sleep (OR = 1.77; 95% CI, 1.31–2.40; *P* < .001) (Table 2). Food-insecure adolescents also had an 81% increased odds of reporting current cigarette smoking compared with food-secure adolescents (OR = 1.81; 95% CI, 1.32–2.49; *P* < .001) (Table 2).

**Table 2:** Univariate Logistic Regression Model Results, Pennsylvania Adolescents, Youth Risk Behavior Survey, 2014–2015

Outcome	Food Secure		Food Insecure	
	AOR (95% CI)	*P* Value	AOR (95% CI)	*P* Value
**Dietary**				
Did not eat breakfast	1 [Reference]^a^		2.46 (1.75–3.47)	<.001
Ate <5 servings per day of fruits and vegetables	1 [Reference]^b^		1.15 (0.78–1.70)	.48
Drank soda 1 or more times/day	1 [Reference]^c^		1.33 (0.97–1.81)	.08
Drank milk <1/day	1 [Reference]^d^		1.02 (0.82–1.27)	.86
**Body weight, physical activity, screen time, sleep**				
Overweight or obese	1 [Reference]^e^		1.18 (0.92–1.52)	.18
Did not do 60 minutes per day of physical activity on all 7 days	1 [Reference]^f^		0.98 (0.75–1.28)	.87
Had 3 hours or more per day of screen time	1 [Reference]^g^		1.34 (0.96–1.86)	.08
Had less than 8 hours per day of sleep	1 [Reference]^h^		1.77 (1.31–2.40)	<.001
**Substance use**				
Currently smoke cigarettes	1 [Reference]^i^		1.81 (1.32–2.49)	<.001
Currently drink alcohol	1 [Reference]^j^		1.39 (1.07–1.80)	.01

Abbreviations: AOR, adjusted odds ratio, CI, confidence interval.

a Ate breakfast on all 7 days.

b Ate 5 or more servings per day of fruits and vegetables.

c Drank soda less than once per day.

d Drank milk one or more times per day.

e Not overweight or obese.

f Did 60 minutes per day of physical activity on all 7 days.

g Had less than 3 hours per day of screen time.

h Had 8 hours or more of sleep per day.

i Did not currently smoke cigarettes.

j Did not currently drink alcohol.

After adjusting all models for sociodemographic characteristics, including sex, grade, race/ethnicity, and neighborhood safety, food insecurity remained siginificantly associated with not eating breakfast daily, insufficient sleep, current smoking, and drinking. Specifically, food-insecure adolescents had a more than twofold increased odds of not eating breakfast daily than food-secure adolescents (AOR = 2.27; 95% CI, 1.61–3.21; *P* < .001) (Table 3). Compared with food-secure adolescents, those who were food insecure had a 60% increased odds of reporting less than 8 hours per day of sleep (AOR = 1.60; 95% CI, 1.15–2.23; *P* = .006) (Table 3). Food insecure adolescents had a 65% increased odds of reporting current cigarette smoking compared with food-secure adolescents (AOR = 1.65; 95% CI, 1.16–2.36; *P* = .006) (Table 3).

**Table 3:** Adjusted^a^ Logistic Regression Model Results, Pennsylvania Adolescents, Youth Risk Behavior Survey, 2014–2015

Outcome	Food Secure		Food Insecure	
	AOR (95% CI)	*P* Value	AOR (95% CI)	*P* Value
**Dietary**				
Did not eat breakfast	1 [Reference]^b^		2.27 (1.61–3.21)	<.001
Ate <5 servings per day of fruits and vegetables	1 [Reference]^c^		1.13 (0.77–1.67)	.52
Drank soda 1 or more times/day	1 [Reference]^d^		1.19 (0.86–1.64)	.29
Drank milk <1/day	1 [Reference]^e^		0.92 (0.71–1.18)	.50
**Body weight, physical activity, screen time, sleep**				
Overweight or Obese	1 [Reference]^f^		1.07 (0.82–1.40)	.61
Did not do 60 minutes per day of physical activity on all 7 days	1 [Reference]^g^		0.90 (0.68–1.19)	.46
Had 3 hours or more per day of screen time	1 [Reference]^h^		1.28 (0.90–1.81)	.17
Had less than 8 hours per day of sleep	1 [Reference]^i^		1.60 (1.15–2.23)	.006
**Substance use**				
Currently smoke cigarettes	1 [Reference]^j^		1.65 (1.16–2.36)	.006
Currently drink alcohol	1 [Reference]^k^		1.36 (1.01–1.84)	.04

Abbreviations: AOR, adjusted odds ratio; CI, confidence interval.

a Models were adjusted for sex, race/ethnicity, school grade, and neighborhood safety.

b Ate breakfast on all 7 days.

c Ate 5 or more servings per day of fruits and vegetables per day.

d Drank soda one or more times per day.

e Drank milk 1 or more times per day.

f Not overweight/obese.

g Did 60 minutes per day of physical activity on all 7 days.

h Had less than 3 hours per day of screen time.

i Had 8 hours or more per day of sleep.

j Did not currently smoke cigarettes.

k Did not currently drink alcohol.

## Discussion

Poor cardiometabolic health is a significant public health issue that is particularly evident in food-insecure adults (2,3). The main findings from this study were that 12.8% of the adolescents in our sample reported food insecurity, which was significantly associated with skipping breakfast, insufficient sleep, current smoking, and current alcohol consumption. These data begin to develop a picture of how food insecurity may be an important determinant of cardiometabolic risk factors in adolescents.

In adolescents, skipping breakfast is common (18,19). Our data show that compared with food-secure adolescents, those who were food insecure were more than twice as likely not to eat breakfast on all 7 days of the week. In previous studies, food insecurity has been negatively associated with breakfast consumption among college freshmen (20), whereas no relationship was found among students in grades 4 to 6 (21). One possible reason for the lack of association between food insecurity and breakfast consumption in younger children may be the greater uptake of the School Breakfast Program in this group (22). Insofar as skipping breakfast is associated with cardiometabolic outcomes such as adiposity, dyslipidemia, and impaired glucose metabolism (23), our data suggest that identifying ways to increase participation in the breakfast programs among high-school–aged adolescents may be beneficial to ameliorating development of cardiometabolic risk factors in food-insecure adolescents.

Our study is the first to show food insecurity to be associated with sleep patterns in adolescents, independent of other demographic factors. Another study that examined the relationship between food insecurity and sleep in a national sample of 4,081 adults showed food insecurity to be significantly associated with sleep deficits, including longer sleep latency, difficulty falling asleep, sleep maintainence difficulties, early morning awakenings, and nonrestorative sleep, in both unadjusted and adjusted models (24). Results from these 2 studies support the hypothesis that the association between food insecurity and sleep is independent of sociodemographic factors and that food insecurity may be a novel socioeconomic indicator of sleep deficits (24).

Our data showing that food-insecure adolescents had a 65% increased adjusted odds of being current smokers compared with food-secure adolescents adds to a growing body of literature on this relationship among adults. For example, previous studies have shown current smoking to be significantly associated with food insecurity in adults (25), even after adjustment for household income (26,27). The association between tobacco use and food insecurity has traditionally been attributed to socioeconomic factors: when low-income households allocate disposable income to purchasing cigarettes, less money remains for the purchase of food than remains for food among nonsmoking households (28). Given that adult studies show that a higher prevalence of current smoking in food-insecure adults may be independent of economics (26,27), other mechanisms may link tobacco use with food insecurity.

That overweight and obesity were not significantly associated with food insecurity in both the unadjusted and adjusted models was surprising. Food insecurity is thought to co-occur with overweight and obesity. Although our data show that the prevalence of overweight and obesity was higher among food-insecure adolescnts, food insecurity was not a significant predictor of overweight or obesity in the model adjusted for sociodemographic characteristics. Furthermore, the prevalence of overweight and obesity in the overall sample was 29.8%, which is slightly lower than the 34.5% of adolescents aged 12 to 19 years reported in the 2011–2012 National Health and Nutrition Examination Survey (NHANES) (29). This difference is probably because adolescents self-reported their height and weight in our study, whereas in NHANES, researchers measured height and weight. The lack of a significant association between food insecurity and overweight and obesity in the adjusted model for our study underscores the complexity of this relationship, which has yet to be clearly defined (30).

These data suggest that adolescents align more with adults than with younger children in terms of the relationship between food insecurity and cardiometabolic risk factors. These results have clinical and public health implications. Screening for food insecurity in the primary care setting is not common practice. Approximately 72.2% of adolescents (12–17 y) had contact with a health care professional in the last 6-months (31) indicating that primary care may be a viable setting in which to identify food insecurity. However, adolescents from low-income families may have limited access to primary health care. On the population level, federal food assistance programs, such as the School Breakfast Program, can have a broad impact. Federal food assistance programs provide low-income adolescents with food, which can have a downstream effect on the cardiometabolic risk factors associated with food insecurity, such as skipping breakfast. Our data suggest that these programs are important, but uptake of these programs is necessary to achieve their intended purpose (eg, alleviate hunger).

A strength of this study is its basis in a large, statewide survey. Data obtained by the YRBS are essential for understanding the behavioral patterns of adolescents in Pennsylvania and in the United States as a whole, and findings can serve as a basis for further analytical research. Study limitations are its cross-sectional study design and that YRBS data were self-reported, which could introduce bias into responses. Also, the classification of food insecurity was based on a single-item question.

Our study contributes to understanding of associations between food insecurity and cardiometabolic risk factors in adolescence. Food insecurity may be an important contributor to the cardiometabolic health profile of an adolescent. Longitudinal studies examining this relationship are needed.
